# Real‐world experience: Introduction of T cell replete haploidentical transplantations in a single center

**DOI:** 10.1002/jha2.203

**Published:** 2021-05-26

**Authors:** Gwendolyn van Gorkom, Evy Billen, Catharina Van Elssen, Michel van Gelder, Gerard Bos

**Affiliations:** ^1^ Division of Hematology Department of Internal Medicine GROW School for Oncology and Developmental Biology Maastricht University Medical Center Maastricht The Netherlands

**Keywords:** haploidentical, hematopoietic stem cell transplantation, posttransplantation cyclophosphamide

## Abstract

**Objectives:**

The aim of this study was to describe real‐world data on outcomes of T cell replete haploidentical hematopoietic stem cell transplantation (HSCT) after the introduction of this modality in a single center and to compare them with different donor types.

**Method:**

Outcomes of 30 consecutive patients with hematological malignancies that received T cell replete haploidentical HSCT with posttransplantation cyclophosphamide (PTCY) from 2016 to 2018 in our center were analyzed and compared to the outcome of human leukocyte antigen (HLA)‐related and unrelated matched donor HSCT (*n* = 97) and to a historical cohort of T cell depleted haploidentical HSCT (*n* = 11).

**Results:**

One year graft‐versus‐host‐free, relapse‐free survival in haploidentical HSCT was comparable with other donor types (haplo 40%, matched related donor [MRD] 33%, matched unrelated donor [MUD] 25%, *p* = 0.55). Non relapse mortality was high in haploidentical HSCT (50%), mostly due to infectious complications. However, relapse rates were only 3%, and OS and progression‐free survival after 1 year were 47% and thereby also similar to HLA‐matched HSCT in our center (MRD 53%, MUD 48%).

**Conclusion:**

Our data show that T cell replete haploidentical HSCT has similar outcomes to HLA identical HSCT after introduction in our center. More strict adaptation on infection prevention was a crucial aspect of our learning curve. Overall, this type of transplantation is a feasible option when lacking an HLA‐identical donor. This option has advantages over an unrelated donor as it brings less logistical challenges than MUD transplantations.

## 1 INTRODUCTION

Allogeneic hematopoietic stem cell transplantation (HSCT) is recommended in some patients with high risk hematological diseases. Historically, the preferred stem cell sources are human leukocyte antigen (HLA) matched related donors (MRD) or 10 of 10 HLA‐matched adult unrelated donors (MUD) in most transplantation centers including ours. Many patients lack such donors. Alternative hematopoietic stem cell donors can be haploidentical donors. The HLA of these donors matches minimally 50% with the HLA type of the patient, and they can often be easily and rapidly found among family members. However, HLA disparity between patient and donor induces a high incidence of severe graft‐versus‐host disease (GVHD) and graft rejection. Special measures are needed to prevent this. One strategy to prevent GVHD is to extensively deplete donor T cells from the graft T cell depleted (TCD). The key publication and subsequent updates from Perugia on haploidentical HSCT with TCD demonstrated that haploidentical HSCT can be effective in patients with acute myeloid leukaemia (AML) and that TCD resulted in an extremely low incidence of GVHD [1, 2, 3]. Non relapse mortality (NRM) with this strategy remained extremely high and resulted mainly from lethal infections due to the prolonged T cell deficient state caused by the extensive TCD by the in vitro CD34+ cell selection. We, and others performed this type of TCD haploidentical HSCT in the past and were not able to duplicate the results from Perugia. We observed an even higher rate of NRM and accordingly extremely low survival rates and have abandoned this type of transplantation [4, 5]. Interesting strategies have been developed to overcome this drawback of TCD haploidentical HSCT, for instance by only removing specific cell types in vitro [6, 7]. Different types of TCD are explained more in detail by Or‐Geva and Reisner [8].

Since the development of in vivo T cell depletion with posttransplantation cyclophosphamide (PTCY), the use of haploidentical donor cells is universally increasing [9, 10]. The biological concept of this technology is to functionally impair alloreactive donor T cells, activated by day 3–5 after transplant, and spare non‐alloreactive T cells [11]. These unmanipulated T cell replete haploidentical HSCTs are characterized by an encouraging overall survival (OS) and low incidences of GVHD and relapse [9, 12–15].

Several retrospective studies indicate that the results of transplantations with haploidentical grafts are comparable to HLA identical grafts [12, 16–19], and perhaps even better than those after an umbilical cord blood (UCB) graft or an MUD graft [1, 12, 18, 20–22]. Even a large meta‐analysis comparing haploidentical HSCT (*n* = 1410 patients) to MRD HSCT (*n* = 6396 patients) showed no significant differences between both treatment groups with regard to OS, relapse, and GVHD‐free, relapse‐free survival (GRFS) [23]. T cell replete haploidentical HSCT has several other benefits: limited costs for the donor transplant (compared to MUD or UCB), rapid availability for almost all patients, and the possibility to collect additional cells for cellular therapy at the time of transplant or thereafter [24].

Given this opportunity, we decided to re‐introduce haploidentical HSCT in our hospital in 2016 for patients without HLA‐identical donors, however this time with PTCY. We decided to retrospectively evaluate the outcomes and compare them with outcomes from HLA‐identical HSCT in the same time frame and with our historical cohort of TCD haploidentical HSCT, as a quality control for our institute to see if our transplant data are comparable to those published.

## 2 METHODS

### 2.1 Patients

This is a retrospective study of 127 consecutive patients (30 T cell replete haploidentical, 36 MRD, 61 MUD) undergoing an allogeneic HSCT between January 1, 2016 and September 21, 2018 at the Maastricht University Medical Center, Maastricht, the Netherlands and a cohort of 11 patients receiving a TCD haploidentical HSCT from 2005 to 2011 in the same center; a procedure we stopped because of the poor clinical results. We excluded UCB transplantations as we performed too little of them to draw any conclusions. All patients signed consent forms allowing analysis and dissemination of their outcome data. The follow‐up and analyses were performed with November 2020 as last data point for all patients.

### 2.2 Donor selection criteria

In our institute the following hierarchy for donor choice applied: first donor choice for every patient was an HLA‐identical donor (MRD or 10/10 MUD) but when not available a haploidentical family donor was chosen. Both patient and donor HLA typing were performed using sequence‐based typing for HLA‐A, ‐B, ‐C, ‐DRB1, and ‐DQB1 loci. In case a haploidentical donor was available, patients were tested for the presence of donor‐specific antibodies (DSAs). When DSAs were present in high levels (mean fluorescence intensity > 4000), the donor was excluded.

### 2.3 Conditioning regimens

#### 2.3.1 Haploidentical transplantations

##### T cell replete

Chemotherapy‐based myeloablative conditioning regimens included thiotepa, busulphan, and fludarabine (TBF) in most of these haploidentical stem cell transplantations according to the myeloablative conditioning regimen used in Genua, Italy [25]. In some indications, a radiotherapy‐based conditioning was used with total body irradiation (TBI) consisting of 10 Gray (Gy) in fractionated doses, combined with fludarabine (Flu‐TBI) ± cyclophosphamide before transplantation.

##### TCD

In all cases a myeloablative conditioning regimen was used that included thiotepa, fludarabine, and TBI (8 Gy).

#### 2.3.2 MRD and MUD

A non‐myeloablative conditioning regimen with a combination of low dose TBI (2 Gy) and fludarabine was most often used in HLA‐identical SCT. Other utilized regimens can be found in Table 1.

**TABLE 1:** Patient characteristics

	T cell replete haplo (*n* = 30)	MRD (*n* = 36)	MUD (*n* = 61)	T cell deplete haplo (*n* = 11)	*P* value
Characteristic	No. (%)	No. (%)	No. (%)	No. (%)	
Median age (range) (years)	60.3 (19–74)	61.3 (23–71)	58.3 (21–76)	43.8 (19–61)	0.002
Sex					0.70
Male	20 (67)	24 (67)	35 (57)	6 (55)	
Female	10 (33)	12 (33)	26 (43)	5 (45)	
HCTCI score					0.26
0	19 (63)	17 (47)	32 (52)	10 (91)	
1 or 2	7 (23)	12 (33)	18 (30)	1 (9)	
≥3	4 (13)	7 (19)	11 (18)	0 (0)	
Diagnosis					0.06
AML	16 (53)	16 (44)	19 (31)	8 (73)	
ALL	6 (20)	3 (8)	6 (10)	0 (0)	
MDS/MPN	6 (20)	5 (14)	16 (26)	1 (9)	
NHL/HL/CLL	2 (7)	8 (22)	16 (26)	0 (0)	
Other	0	4 (11)	4 (7)	2 (18)	
Disease risk index					0.34
Low	0 (0)	5 (14)	4 (7)	0 (0)	
Int	17 (57)	19 (53)	36 (59)	7 (64)	
High	13 (43)	12 (33)	21 (34)	4 (36)	
Number allogeneic transplantation					0.33
First	29 (97)	33 (92)	58 (95)	9 (82)	
Second	1 (3)	3 (8)	3 (5)	2 (18)	
CMV status recipient/donor					0.90
+/−	10 (33)	10 (28)	17 (28)	4 (36)	
Other	20 (67)	26 (72)	44 (72)	7 (64)	
Recipient/donor sex match					0.009
M/F	10 (33)	13 (36)	6 (10)	2 (18)	
Other	20 (67)	23 (64)	55 (90)	9 (82)	
Donor age, years					<0.001
≤40	15 (50)	4 (11)	52 (85)	5 (45)	
> 40	15 (50)	32 (89)	9 (15)	6 (55)	
Regimen type					<0.001
MAC	29 (97)	10 (28)	16 (26)	11 (100)	
Bu‐based	27	6	10	0	
TBI‐based	2	3	6	11	
Other	0	1	0	0	
RIC	1 (3)	10 (28)	14 (23)	0	
Flu/Cy	1	6	11	0	
Cy/TBI	0	2	1	0	
Other	0	2	2	0	
NMA	0 (0)	16 (44)	31 (51)	0	
Flu/TBI	0	16	31	0	
Stem cell source					<0.001
PBSC	3 (10)	35 (97)	58 (95)	0 (0)	
BM	27 (90)	1 (3)	3 (5)	11 (100)	

### 2.4 Stem cell source

The preferred source of stem cells in T cell replete haploidentical HSCT was bone marrow (BM). Peripheral blood (PB) was preferably used in HLA‐identical transplantations. In these transplantations, unmanipulated BM and PB stem cells were given on day 0. Only in cases with ABO major or minor incompatibilities, red blood cell or plasma depletion of the harvested product was performed.

In TCD haploidentical HSCT, PB stem cells were used after ex vivo CD34 selection and cryopreservation.

### 2.5 GVHD prophylaxis

In recipients of T cell replete haploidentical grafts PTCY 50 mg/kg intravenously on days +3 and +5 was given to create in vivo T cell depletion, and they received cyclosporine (CyA) from day 0 until day +180 and mycophenolate mofetil (MMF) from day +1 to day +28 [25].

Most recipients of HLA‐identical stem cell transplantations received CyA from day −3 to day +180 and MMF from day +1 to day +85. Only patients diagnosed with myelofibrosis also received anti thymocyte globulin (ATG).

Recipients of TCD haploidentical grafts received ATG on days −6 to −2, but no GVHD prophylaxis after stem cell transplantation.

### 2.6 Supportive care

During neutropenia ciprofloxacin and fluconazole were given as selective digestive tract decontamination. Anti‐microbial prophylaxis furthermore consisted of valacyclovir, and after 1 year experience on T cell replete haploidentical HSCT cotrimoxazole was added to prevent pneumocystis infections.

### 2.7 Endpoints

The primary endpoint was GRFS at 1 and 2 year. This was defined as time from transplantation until grade III‐IV acute GVHD (aGVHD), chronic GVHD (cGVHD) requiring systemic immunosuppressive treatment, disease relapse or death, whichever occurred first [26]. Secondary endpoints were OS, progression‐free survival (PFS), relapse rate, NRM, incidence and severity of aGVHD and cGVHD and time to engraftment.

### 2.8 Statistical method

Categorical variables are expressed as number and proportion, and continuous variables as median and range. The Kaplan–Meier method was used for the OS, PFS, and 1‐year GRFS analyses.

The cumulative incidences of acute and chronic GVHD were estimated considering death not related to GVHD as a competing event. For the calculation of NRM, disease relapse or progression was treated as a competing event, and for the calculation of relapse, NRM was treated as a competing event. Outcomes were calculated from the day of transplantation. Comparisons between all groups were made using log‐rank and Gray tests, and *p*‐values of these comparisons were given.

Analyses were performed using SPSS, version 25, and R software.

## 3 RESULTS

### 3.1 Patients

Patient characteristics are summarized in Table 1.

In the T cell replete haploidentical group, the median age at transplant was 61 years, 70% of patients were older than 55 years. The most common diagnosis was AML (53%). The other groups (MRD, MUD, TCD haploidentical) were well matched for the indicated demographics, with two exceptions: patients in the TCD haploidentical group were younger and in that group, there were hardly any other transplant indications than AML (73%). Most patients were in remission at the time of transplantation, and there were no significant differences between the treatment groups.

Most T cell replete patients received a TBF‐based, myeloablative conditioning regimen (97%), while this was only around 27% in HLA‐matched transplantation. In 90% of the T cell replete haploidentical HSCT, the stem cell source was BM. MUD donors were generally younger and better sex matched.

Median time to follow‐up of patients alive was 37.5 months for T cell replete haploidentical HSCT, 36.8 months for MRD HSCT, and 46.0 months for MUD HSCT.

### 3.2 Outcomes of T cell replete haploidentical HSCT

**FIGURE 1:**
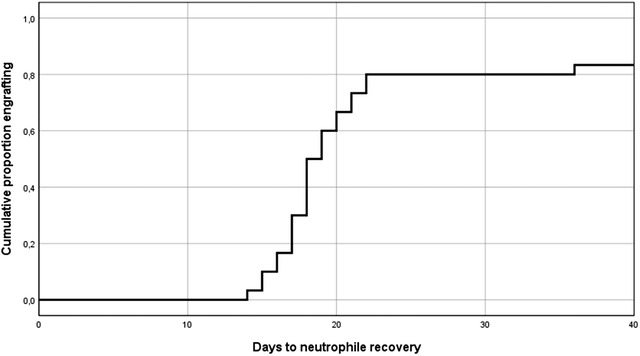
Neutrophil recovery after T cell replete haploidentical HSCT

Median time to neutrophil engraftment was 18 days (range 14–36) (Figure 1). Five patients (17%) did not show engraftment, most of these patients died before engraftment of infectious complications. In univariate analysis, in this small group there was no correlation between the number of nucleated cells present in the graft and engraftment.

**FIGURE 2:**
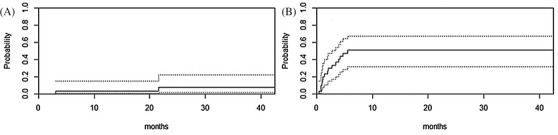
Cumulative incidence of non‐relapse mortality (NRM) and relapse in T cell replete haploidentical HSCT. (A) Relapse after stem cell transplantation (with 95%CI). (B) NRM after stem cell transplantation (with 95% CI)

After a median follow‐up of 37.5 months (range, 25.9–54.5), 13 of 30 patients were alive and in remission. The 1‐year OS and PFS was 47% (95% confidence interval [CI], 30–64), and 2‐year OS and PFS 43% (95% CI, 26–60), with a 1‐year relapse incidence of 3% (95% CI 0–9) (Figure 2A). NRM at 1 year was 50% (95% CI 31–67) (Figure 2B). Causes of death in the first year are listed in Table 2. The main cause of death was infection (86%). Details about the type of infections can be found in Table 2.

**TABLE 2:** Causes of death after T cell replete haploidentical HSCT

Cause of death	Number of patients
Sepsis	7
Pneumocystis jirovecii pneumonia	3
Viral respiratory infection	3 (1 corona NL63, 2 para influenza type 3)
Aspergillus infection	1
Graft failure	1
Relapse	1

In multivariate analysis (looking at age, gender, disease risk index, HCT‐CI, recipient‐donor sex match, recipient‐donor CMV status, donor age), patient age was the only factor that correlated with OS (hazard ratio 2.4). GRFS at 1 and 2 year was 40% (95% CI 22–58) with no new events after 1 year.

**FIGURE 3:**
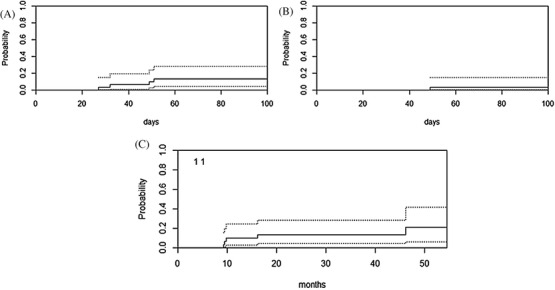
Cumulative incidence of GVHD in T cell replete haploidentical HSCT. (A) Grades 2–4 acute GVHD (with 95% CI). (B) Grades 3–4 acute GVHD (with 95% CI). (C) All chronic GVHD (with 95% CI)

The cumulative incidences of grade II–IV and grade III–IV aGVHD at 100 days post‐transplant were 13% (95% CI 4–28) and 3% (95% CI 0–15), respectively (Figures 3A and 3B). The cumulative incidence of cGVHD at 1‐ and 2‐years post‐transplant was 10% (95% CI 2–24) and 13% (95% CI 4–28), respectively (Figure 3C).

### 3.3 Comparison with outcomes of MRD and MUD HSCT

**FIGURE 4:**
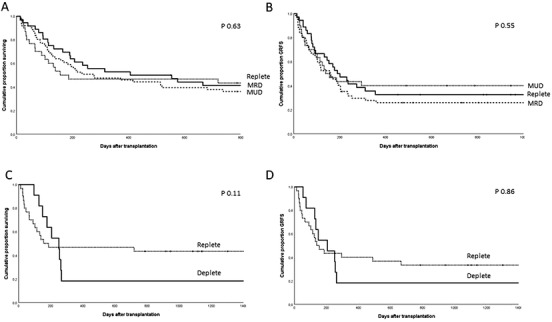
Adjusted estimated probabilities of overall survival (OS) and GVHD‐free relapse‐free survival (GRFS) in T cell replete haploidentical HSCT and all donor types. (A) OS T cell replete haploidentical versus MRD versus MUD HSCT. (B) GRFS T cell replete haploidentical versus historical T cell deplete haplo HSCT. (C) OS T cell replete haploidentical versus historical T cell deplete haploidentical HSCT. (D) GRFS T cell replete haploidentical versus MRD versus MUD HSCT

There were no significant differences between the outcomes of HSCT with a T cell replete donor, MRD, or MUD. One year OS rates were 47% (95% CI 30–65), 53% (95% CI 37–69), and 48% (95% CI 35–61) for T‐cell replete haploidentical, MRD, and MUD (*p* = 0.63) (Figure 4A). One year relapse incidence was also similar in T cell replete haploidentical HSCT to MRD and MUD, respectively 3% (95% CI 0–9), 17% (95% CI 5–29), and 21% (95% CI 11–21) (*p* = 0.08). NRM at 1 year was also not significantly different between these groups, respectively 50% (95% CI 31–67) for T cell replete haplo, 33% (95% CI 17–49) for MRD, and 36% (95%CI 24–48) for MUD, (*p* = 0.43). The cumulative incidences of grade II–IV and grade III–IV aGVHD at 100 days post‐transplant were not significantly different as well, but there was a trend toward a higher probably of aGVHD in MUD HSCT with a grade II–IV of 23% (95% CI 13–34) and grade III–IV 16% (95% CI 8–27) versus 13% (95% CI 4–28) and 3% (95% CI 0–15) in T cell replete haploidentical HSCT and 8% (95% CI 2–20) and 6% (95% CI 0–17) in MRD HSCT, respectively (*p* = 0.08 and *p* = 0.09). The cumulative incidence of cGVHD at 1‐ and 2‐years post‐transplant were 10% (95% CI 2–24) and 13% (95% CI 4–28) for T cell replete haploidentical HSCT, 11% (95% CI 4–25) and 19% (95% CI 8–34) for MRD HSCT and 23% (95% CI 13–34) and 25 (95% CI 16–36) for the MUD HSCT, respectively (*p* = 0.32).

One‐ and 2‐year GRFS rates were also similar (40% (95% CI 22–58), 33% (95% CI 17–49), and 26% (95% CI 14–38) for T cell replete haploidentical, MRD, and MUD, respectively (*p* = 0.55) (Figure 4B)).

### 3.4 Comparison with outcomes of TCD haploidentical HSCT

One year OS and GRFS was better in T cell replete haploidentical HSCT than in the historical TCD haploidentical HSCT cohort (though not significant due to low numbers); OS was 47% (95% CI 30–65) and 18% (95% CI 0–41) (*p* = 0.11), respectively, and GRFS was 40% (95% CI 22–58) and 18% (95% CI 0–41), respectively (Figures 4C and 4D) (*p* = 0.86).

## 4 DISCUSSION

T cell replete haploidentical HSCT with PTCY is increasingly used worldwide, but data shown in literature are mostly from large multicenter analyses and generally from centers with high volumes. Here, we report the introduction of this transplantation method in our relatively small transplantation center, which is the smallest in the Netherlands [27]. On average we perform 40–50 allogeneic HSCT per year, with a staff of only nine hematologists.

In 2016, we introduced haploidentical HSCT combined with PTCY. In this retrospective analysis for quality control ‐ we find OS and GRFS of T cell replete haploidentical HSCT to be like those of our MRD and MUD HSCT.

Remarkably, NRM rate was much higher than expected from literature. In large retrospective registry studies, NRM is 10%–25%, while in our patients this was 50% [28, 29, 30, 31]. The main cause of death was infectious complications after transplantation. This could be partly due to an unfortunate and serious outbreak of respiratory viral infections at our ward during this period. Of the 15 patients that received a T cell replete haploidentical HSCT during this outbreak, seven patients were diagnosed with a respiratory viral infection, and three died due to this infection. After this experience, in our second year of T cell replete haploidentical HSCT, we adapted our policy to prevent viral infections on the ward (for instance constant wear of mouth masks, limitation of visitors, early extensive testing, stricter isolation). After all these measurements, we noticed a decrease in incidence of infections (only one of 15 patients was diagnosed with a respiratory viral infection) and a decreased mortality (no patient died of respiratory viral infections in this time period).

Another reason for the high NRM rate could be the high incidence of pneumocystis jivorecii pneumonia (PJP) (five of 15 patients in the first year). After 1 year of performing haploidentical T cell replete HSCT, we started to use prophylactic cotrimoxazole and noticed a decrease in PJP incidence (one of 15 patients).

After all these adaptations, NRM decreased from 60% to 40%. This is still quite high but could partly be explained by the fact that most of the patients were of older age (median 60.3 years) and were all still receiving myeloablative conditioning. Recently after this analysis, we adapted our policy and decided to use a reduced intensity conditioning regimen in older patients to see if this could lower the mortality rate. However, this might be at the cost of a higher risk of relapse.

In other retrospective studies, a prominent cause of death was disease relapse (with 25%–45% of patients experiencing relapse within 2 years) [10, 28–31]. Only one of our patients (3%) experienced relapse in the first year in the T cell replete haplo group, so our relapse rate was much lower than expected. This could be partly explained by the high NRM that is a competing risk for relapse and could also be linked to the myeloablative conditioning regimen.

Cumulative incidences of acute and chronic GVHD were low in our haploidentical T cell replete HSCT, and comparable to those in literature when BM is used as stem cell source [28, 29, 30, 31].

Due to the low relapse and GVHD rate, OS, PFS, and GRFS were comparable to outcomes found in literature. However, direct comparisons are difficult to be made since our numbers are limited and because these outcomes are dependent of different risk factors like age, co‐morbidity, and disease status.

In our center, HLA‐identical HSCT at time of the analysis was still performed without PTCY. The use of PTCY in HLA‐identical HSCT can potentially improve the outcome. In a recent randomized trial a 1‐year GRFS in HLA‐identical transplantations (MRD and MUD) of 45% was shown, but this is in the same range as our data with haploidentical HSCT [32]. Another option to improve GRFS in HLA‐identical HSCT could be the addition of ATG, as we did not use this in most of our patients. However, in most studies ATG only lowers GVHD and does not impact OS [33, 34] and gives similar results to PTCY [35].

In the past, we performed some TCD haploidentical HSCT using the same method as described by the Perugia group. In contrast to their results, we had an extremely high NRM rate that was mainly due to infections, and we noticed that less than a fifth of patients survived. We hypothesize that the difference in climate in the Netherlands versus Italy, and perhaps also of living conditions, might be the reason for that. Also other centers could not reproduce the good outcomes of the Perugia group so these cannot be the only factors responsible for the worse outcome. Therefore, in our hands, and in those of others, this type of TCD haploidentical HSCT was not seen as a good alternative for patients lacking an HLA‐identical donor, and after this experience we stopped performing haploidentical HSCT for several years. Perhaps, these serious infectious problems could have been partly avoided with a different type of TCD. In the CD34 positive cell selection not only all T cells, but also natural killer cells and B cells are removed, while in other types of in vitro TCD‐specific subsets are removed and thereby the immune system remains more intact.

For the T‐cell replete haploidentical transplantations that were performed more recently, we see better outcomes than in our historical patient group.

In light of this analysis and the logistical problems we experienced last year due to the COVID‐19 pandemic, we recently even adapted our search strategy and are preferring a haploidentical donor above an MUD 10 of 10 when there are no DSAs.

## 5 CONCLUSION

In summary, this comparison shows that outcomes after T cell replete haploidentical HSCT are comparable to those of HLA‐identical HSCT in our hands. They are better than the outcomes of TCD haploidentical HSCT in our center in the past. We performed this study as quality control for our institute to see if our transplant data are comparable to those published. At the start, we experienced a learning curve but could decrease our initially remarkably high TRM due to infectious complications. Even though this study has its limitations due to the small number of patients, the heterogeneity between them and the retrospective nature, we conclude that the use of a haploidentical donor is a valid real‐world choice for patients in need for an allogeneic HSCT. A haploidentical donor is usually quickly available for almost all patients, and the choice for this type of donor is logistically easier to arrange than an MUD.

## CONFLICT OF INTEREST

The authors declare no relevant conflict of interest.

## Data Availability

The data that support the findings of this study are available from the corresponding author upon reasonable request.
